# Three-dimensional fusion imaging to assess apposition of low-profile visualized intraluminal support stent for intracranial aneurysm coiling

**DOI:** 10.1016/j.wnsx.2024.100381

**Published:** 2024-04-21

**Authors:** Naoki Kato, Toshihiro Ishibashi, Katharina Otani, Yukiko Abe, Tohru Sano, Gota Nagayama, Michiyasu Fuga, Shunsuke Hataoka, Issei Kan, Yuichi Murayama

**Affiliations:** a Department of Neurosurgery, The Jikei University School of Medicine Tokyo, Tokyo, Japan; b Siemens Healthcare K.K., Tokyo, Japan; c Department of Radiology, The Jikei University Hospital, Tokyo, Japan

**Keywords:** Aneurysm, Angiography, C-arm, Coiling, Computed tomography, Stent

## Abstract

**Objective:**

To investigate on three-dimensional (3D) fusion images the apposition of low-profile visualized intraluminal support (LVIS) stents in intracranial aneurysms after treatment and assess inter-rater reliability.

**Materials and methods:**

Records of all patients with unruptured intracranial aneurysms who were treated with the LVIS stent were retrospectively accessed and included in this study. Two neurosurgeons evaluated the presence of malapposition between the vessel walls and the stent trunk (crescent sign) and the vessel wall and the stent edges (edge malappostion) on 3D fusion images. These images were high-resolution cone-beam computed tomography images of the LVIS stent fused with 3D-digital subtraction angiography images of the vessels. Associations between malapposition and aneurysm location were assessed by Fisher's exact test, and inter-rater agreement was estimated using Cohen's kappa statistic.

**Results:**

Forty consecutive patients were included. In all patients, 3D fusion imaging successfully visualized the tantalum helical strands and the closed-cell structure of the nitinol material of the low-profile visualized intraluminal support. A crescent sign was observed in 27.5 % and edge malapposition in 47.5 % of the patients. Malapposition was not significantly associated with location (*p* = 0.23 crescent sign, *p* = 0.07 edge malapposition). Almost perfect (*κ* = 0.88) and substantial (*κ* = 0.76) agreements between the two raters were found for the detection of crescent signs and edge appositions, respectively.

**Conclusions:**

3D fusion imaging provided clear visualization of the LVIS stent and parent arteries, and could detect malapposition with excellent inter-rater reliability. This technique may provide valuable guidance for surgeons in determining postoperative management.

## Research institute

Department of Neurosurgery, The Jikei University School of Medicine Tokyo.

## Grant support

This study was partially supported by a research grant (ID C00221026) from 10.13039/501100004830Siemens Healthcare K.K.

## 1 Introduction

Low-profile visualized intraluminal support (LVIS) stents have been widely used for stent-assisted coiling of intracranial aneurysms because of their post-treatment safety and high occlusion rate.^1^ Ensuring optimal stent apposition is an important factor for preventing thromboembolic complications for all types of stents.^2^ Stent apposition is commonly evaluated on two-dimensional (2D) maximum intensity projection (MIP) images acquired by the angiographic C-arm system after the injection of low-concentration iodinated contrast medium.^3^^,^^4^ Recent studies have reported an advanced imaging protocol for stent visualization after coiling, surpassing the more traditional 2D MIP imaging method in precision.^5^ It involves fusing high-resolution cone-beam computed tomography (HR-CBCT) of the stent and three-dimensional (3D) digital subtraction angiography (DSA) images of the vessels to visualize the precise structure of a stent inside the parent artery.^6^

This technique has been applied to evaluate the apposition of laser-cut stents and flow diverters with excellent inter-rater reliability.^7^^,^^8^ However, the LVIS stent, a braided stent indicated for coil-assisted stenting, features a body that consists of nickel-titanium (nitinol) material in a closed-cell construction, including two to three tantalum helical strands within the body of the stent as radiopaque markers. We predicted that the presence of these radiopaque markers would pose a challenge when attempting to visualize the LVIS stent.^9^ Thus, in this study, we aimed to apply the 3D fusion imaging technique to evaluate stent apposition in patients with intracranial aneurysms who underwent stent-assisted coiling using LVIS stents and to investigate the proportion of stent malapposition.

## 2 Methods

### 2.1 Patient population

This study was reviewed and approved by the Institutional Review Board (IRB) of our university hospital [reference # 27–236 (8121)]. Informed consent for stent-assisted coiling and CBCT acquisition was obtained from all patients. The IRB waived the requirement for informed consent for this study because the data were obtained from routine procedures and were retrospectively analyzed. However, the IRB requested that we post a study notice describing the research and offer patients the opportunity to decline participation.

We retrospectively identified all patients treated for intracranial aneurysms with an LVIS stent (LVIS and LVIS Jr.; MicroVention-Terumo, Tustin, CA, USA) at our institution between August 2016 and December 2022. All patients had been treated with stent assisted coiling using the jailing technique.

### 2.2 Imaging

**Fig. 1:**
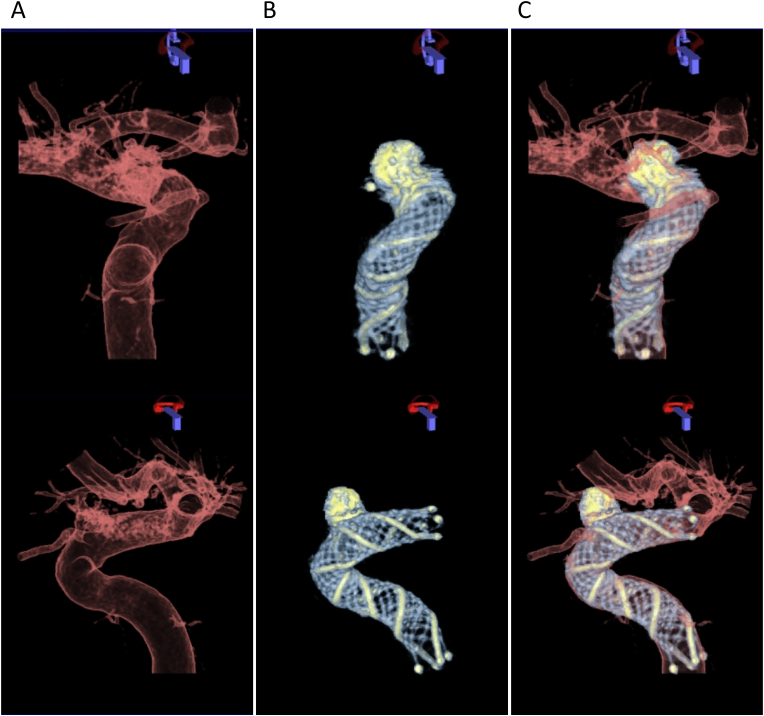
Step-by-step reconstruction of dual-volume three-dimensional (3D) fusion images. 3D vessel images acquired from 3D digital subtraction angiography. (A) (Upper) anterior-posterior and (lower) lateral view. 3D images of low-profile visualized intraluminal support (LVIS) stent generated from high-resolution cone-beam computed tomography. (B) (Upper) anterior-posterior and (lower) lateral view. Dual-volume 3D fusion images created from the 3D vessel images and the 3D stent images. (C) (Upper) anterior-posterior and (lower) lateral view.

All endovascular treatments and follow-up procedures were performed using a biplane angiographic C-arm system (Artis Q Biplane, Siemens Healthcare GmbH, Forchheim, Germany). 2D-DSA images were acquired at a frame rate of 4 fps after the injection of a contrast material (Optiray, ioversol 320 mg I/mL, Fuji Pharma, Tokyo, Japan; Visipaque, iodixanol 270 mg I/mL, GE HealthCare Pharma, Tokyo, Japan; Iohexol, iohexol 300 mg I/mL, Hikari Pharmaceutical, Tokyo, Japan) diluted 2:1 with saline solution. During the procedure, the neurosurgeons applied an imaging protocol to confirm stent apposition following a previous report.^6^^,^^7^ Patients underwent a 6-s 3D-DSA scan, which consisted of a mask and a fill run acquisition (tube voltage of 70 kVp, dose of 0.36 μGy/Fr, scan of 260°). The fill run was triggered without delay by injecting undiluted contrast medium (Optiray, ioversol 320 mg I/mL; Visipaque, iodixanol 270 mg I/mL; Iohexol, iohexol 300 mg I/mL) at a rate of 3 mL/s for 6 s (Fig. 1A). Subsequently a 20-s HR-CBCT scan without contrast injection was performed (70 kVp, dose of 1.2 μGy/fr, scan of 200°, nonbinned mode) (Fig. 1B). The data were transferred to a commercial workstation (*syngo* X Workplace; Siemens Healthcare GmbH) for reconstruction and postprocessing. A streak metal artifact reduction algorithm (SMART; Siemens Healthcare GmbH) was applied to reconstruct the HR-CBCT images of the aneurysms if the coil mass was large and the metal artifact was severe.^5^ A translucent preset was applied to the 3D-DSA images, and a customized preset was applied to the HR-CBCT images for optimized visualization of the stent struts. The imaging fusion application of the workstation (*syngo* 3D/3D Fusion; Siemens Healthcare GmbH) subsequently generated dual-volume 3D fusion images from the 3D-DSA images of the vessels and HR-CBCT images of the LVIS stent (Fig. 1C).^5^^,^^6^ Misalignment was minimized by the automatic registration included in the fusion application. The radiation dose report was automatically generated by the system.

### 2.3 Measurement of stent apposition and aneurysm size

**Fig. 2:**
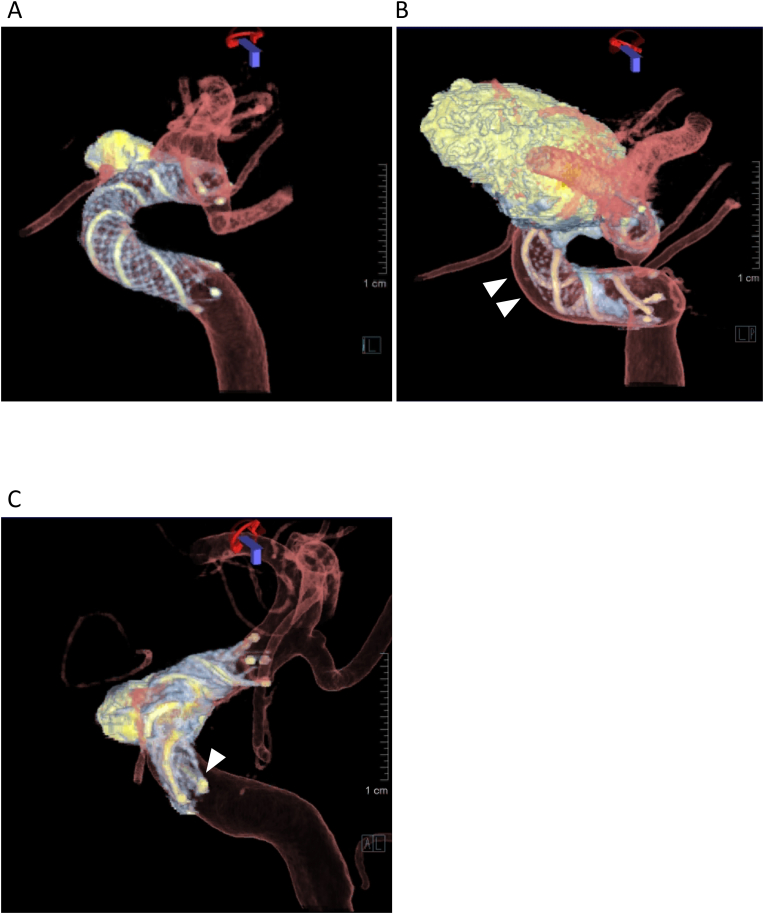
Representative images. (A) Dual-volume three-dimensional (3D) fusion images show good stent apposition within the siphon of the left internal carotid artery (ICA) after stent-assisted coiling using low-profile visualized intraluminal support (LVIS). (B) Crescent sign between the stent trunk of an LVIS stent and the anterior wall of the ICA siphon is clearly displayed (arrow heads). (C) Edge malapposition of proximal flare of a LVIS stent is shown (arrow).

Stent apposition was retrospectively assessed on the 3D fusion images by two board-certified neurosurgeons who were also certified as endovascular surgeons (XX with 19 years and XX with 28 years of experience). The neurosurgeons were blinded to each other's results and the patient's clinical history. They examined the apposition and the location of maximum malapposition using the rotation, zoom, and pan functions, while also being allowed to change the windowing. Fig. 2A shows an example of good stent apposition. We defined malapposition as a lack of contact between the vessel wall and at least one segment of a closed-cell structure made of nitinol with tantalum helical strands or stent flares. Then we determined two types of malapposition according to the following criteria^1^: “crescent sign,” which described a gap between the trunk of the stent and the parent artery wall^10^ (Fig. 2B), and^2^ “edge malapposition,” which described a gap between the distal or proximal flare of the stent and the parent artery^7^ (Fig. 2C). The proportion of stent malapposition was estimated for each aneurysm location. Associations between malapposition and stent features were also analyzed.

Age, sex, and postoperative course were retrospectively retrieved from the patients’ medical records. The size and neck length of the aneurysms were measured on 2D images of DSA acquired before the procedure and the diameter and length of the used stents, volume embolization ratio (VER),^11^ and Raymond–Roy occlusion classification (class 1: complete obliteration, class 2: residual neck, class 3: residual aneurysm)^12^ were obtained from the operative records.

### 2.4 Statistical analysis

Fisher's exact test was used to test an association between the occurrence of malapposition and the aneurysm location. Mann–Whitney's *U* test was used to examine associations of malapposition with stent diameter and length. Statistical significance was defined as *p* < 0.05. The inter-rater agreement for detecting malapposition was estimated using Cohen's kappa (κ). Statistical analyses were performed using commercial software (Stat-View version 5.0, SAS Institute, NC, USA; StataCorp LLC, College Station, TX, USA; and Excel 2019, Microsoft, Redmond, WA, USA).

## 3 Results

A total of 42 patients were reviewed. LVIS stents measuring 2.5 and 5.5 mm in diameter, and 17 and 32 mm in length, were used in all patients for coiling. All the patients underwent 3D fusion imaging. Of these, the 3D fusion images of two patients were excluded because of image artifacts or fusion errors, and the remaining 40 patients were analyzed. The excluded patients had aneurysms in the internal carotid artery (ICA) (*n* = 1) and in the vertebral artery (VA) (*n* = 1). Patient and aneurysm characteristics are shown in Table 1. By location, 75 % of treated brain aneurysms were in the carotid siphon, and the other locations included the anterior cerebral artery, VA, posterior inferior cerebellar artery, and basilar artery. Although the image acquisition process caused no side effects, one patient experienced a stent-related complication (postoperative ischemic stroke). Detailed information regarding the stent characteristics and treatment outcomes are shown in Table 2.

**Table 1:** Patient and aneurysm characteristics.

	Count (%) or mean value (± SD)
Sample size	40
Age (years)	57.8 ± 11.4
Women	29 (72.5)
Location of the aneurysm	
ICA	30 (75)
Other vessels	10 (25)
ACA	1 (2.5)
VA	6 (15)
PICA	1 (2.5)
BA trunk	1 (2.5)
BA top	1 (2.5)
Maximum diameter of the aneurysm (mm)	7.4 ± 3.4
Neck length of the aneurysm (mm)	6.4 ± 4.1

ACA, anterior cerebral artery; BA, basilar artery; ICA, internal carotid artery; PICA, posterior inferior cerebellar artery; SD, standard deviation; VA, vertebral artery.

**Table 2:** Stent Features and Treatment outcomes.

	Count (%) or mean value (± SD)
VER (%)	29.7 ± 8.7
Raymond–Roy class	
1	19 (47.5)
2	11 (27.5)
3	10 (25)
Stent diameter (mm)	4.0 ± 0.6
Stent length (mm)	21 ± 4.7
Stent-related complication	1 (2.5)

SD, standard deviation; VER, volume embolization ratio.

3D fusion imaging was used to visualize the precise structure and location of the LVIS stent in the parent vessels in all 40 patients, even for aneurysms located near the skull base. Although large coil mass or delivery catheter caused some artifacts and affected image quality, all 3D fusion images were diagnostic and the 3D fusion images revealed two or three tantalum helical strands as radiopaque markers, as well as the closed-cell structure of the nitinol material, which are relatively invisible in 2D images. The mean radiation dose for the additional 3D fusion imaging was 61.6 ± 12.9 mGy for the mask run, 61.7 ± 13.0 mGy for the fill run of the 3D-DSA scan, and 323 ± 34.7 mGy for the HR-CBCT scan.

The crescent sign was observed in 11 of 40 patients (27.5 %) and edge malapposition in 19 of 40 patients (47.5 %). By aneurysm location, the crescent sign was found in 10 of 30 patients (33 %) for stents in the ICA and in 1 of 10 patients (10 %) for stents in other vessels. Edge malapposition was found in 17 of 40 patients (56 %) for stents in the ICA and in 2 of 10 patients (20 %) for stents in other vessels. The differences in the occurrence of the crescent sign and edge malapposition according to the location did not reach statistical significance (*p* = 0.23 and *p* = 0.07, respectively) (Table 3). Associations between presence of the malapposition and stent diameter and length were not observed (crescent sign for stent diameter and length; *p* = 0.11 and *p* = 0.17, edge malapposition for stent diameter and length; *p* = 0.18 and *p* = 0.32).

**Table 3:** Stent malapposition according to the aneurysm location.

	Total	ICA	Other vessels	*P* value
Crescent sign	11/40 (27.5)	10/30 (33.3)	1/10 (10)	0.23
Edge malapposition	19/40 (47.5)	17/40 (56.7)	2/10 (20)	0.07

The values are presented as counts (%). ICA: internal carotid artery.

Other vessels included the anterior cerebral artery (*n* = 1), vertebral artery (*n* = 6), posterior inferior cerebellar artery (*n* = 1), trunk of the basilar artery (*n* = 1), and top of the basilar artery (*n* = 1).

Almost perfect agreement (*κ* = 0.88) between the two observers was found for the detection of crescent signs, and substantial agreement (*κ* = 0.76) was found for the detection of edge malapposition.

### 3.1 Illustrative cases

#### 3.1.1 Case 1

**Fig. 3:**
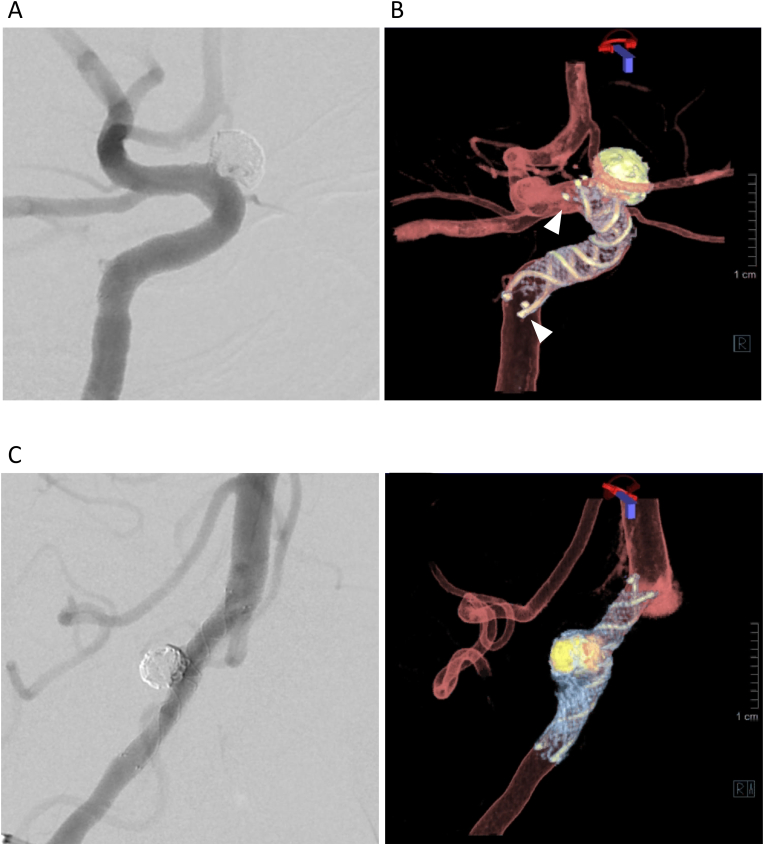
Illustrative images of stent apposition. (A) Case 1: A low-profile visualized intraluminal support (LVIS) stent (4 mm × 22 mm) was deployed for stent-assisted coiling of internal carotid artery aneurysm. (B) Edge malapposition of both the proximal and distal flare of LVIS is clearly visualized (arrowheads) in contrast to the two-dimensional (2D) image. (C) Case 2: An LVIS stent (3.5 mm × 22 mm) was deployed for stent-assisted coiling of vertebral artery aneurysm. (D) All stent struts and edge markers of an LVIS stent are seen, and good apposition of all stent struts was confirmed.

A patient with an unruptured left ICA aneurysm (supraclinoid portion; maximal diameter, 5 mm) was treated with an LVIS stent (4 × 22 mm) (Fig. 3A). The 3D fusion images after stent deployment clearly showed the structures of both the stent and parent artery. Good wall apposition of the stent trunk was seen, but edge malapposition of the distal and proximal flares was detected, which was not visible on the 2D image (Fig. 3B).

#### 3.1.2 Case 2

A patient with an unruptured right vertebral artery aneurysm (maximal diameter, 5.2 mm) was treated with an LVIS stent (3.5 mm × 22 mm) (Fig. 3C). After stent deployment, 3D fusion images revealed the stent structure containing the two tantalum helical strands, as well as the closed-cell structure of nitinol, and confirmed good wall apposition of the stent (Fig. 3D).

## 4 Discussion

In this study, we evaluated stent apposition after LVIS stent deployment on 3D fusion images acquired by a biplane angiographic C-arm system. This imaging technique allowed the surgeons to see the precise structure of the LVIS stent and to confirm stent apposition within their routine workflow. Furthermore, it showed excellent inter-rater agreement for detecting LVIS stent malapposition.

The relatively new-generation LVIS stent is a braided stent with a metal coverage rate between traditional laser cut stents and flow diversion devices. It produces stronger hemodynamic effects and flow reductions on cerebral aneurysms than those of traditional laser cut stents.^13^ The LVIS stent has a closed-cell structure made of nitinol and it also includes a couple of tantalum helical strands for better visibility during the procedure, as it can be challenging to recognize nitinol on 2D images.^9^ Previous studies on three types of laser-cut stents: Neuroform EZ, Neuroform Atlas, and Enterprise 2, have shown that 3D fusion imaging can visualize these stents, even if their main material is nitinol.^7^ For the LVIS stent, we expected that the helical strands included as radiopaque markers would create metal artifacts in the images that would cover the nitinol components or that the 3D fusion images would only visualize the helical strands. However, HR-CBCT successfully reconstructed 3D images of the structure of both the tantalum helical strands and nitinol components.

Stent-assisted coiling is a crucial treatment for wide-necked intracranial aneurysms, and stent apposition is a critical factor in evaluating treatment success.^3^^,^^14^ If malapposition is detected, antiplatelet therapy may need to be adjusted, because incomplete stent apposition can lead to delayed ischemic events after treatment.^3^ Moreover, stent malapposition, particularly at the edges of the stent, can disturb smooth catheterization of the microcatheter tip through the true lumen of the stent during follow-up treatment.^15^ In our study, we found a crescent sign in one-quarter of patients and edge malapposition in approximately half of all patients with LVIS stents. For the ICA aneurysms, although the statistical tests did not reach significance, we observed their tendency towards a larger number of malappositions than in other vessels which might be related to the angled and tortuous anatomical peculiarity of the carotid siphon. Further studies with larger numbers of patients are needed to investigate this trend. In comparison, a previous study found that 27 % Enterprise2 stents had a crescent sign and 27 % had edge malapposition; 8 % Neuroform EZ stents had a crescent sign and 58 % had edge malapposition; and 30 % Neuroform Atlas stents had edge malapposition.^7^^,^^8^

In another study, 3D fusion imaging was used to evaluate the apposition of Pipeline flow diverter stents. That study showed an association between a higher incidence of incomplete apposition and larger stent sizes.^8^ In an animal study, better apposition of flow diverters has also been shown to be associated with more complete occlusions of saccular aneurysms on histological examination.^16^ Similarly, as advanced skills and appropriate manipulation are required for the successful deployment of LVIS stents, the assessment of stent apposition using fusion images would be valuable. In a future study, the association between the apposition of LVIS stents and the proportion of aneurysm recanalization after stent-assisted coiling might be investigated. Larger studies are important, as with larger patient numbers, we may also be able to assess the incidence of thromboembolic events in patients with stent malapposition. Other future studies could include comparisons of the present imaging method with other imaging techniques to evaluate stent malapposition rates.

Various approaches have been documented to identify stent malapposition. While magnetic resonance imaging (MRI) using contrast agents can assess both the stent structure and the parent artery, its intraoperative application may be limited because of the absence of an angiographic suite equipped with an MR scanner.^17^ Conversely, optical coherence tomography (OCT) has been suggested as an alternative; however, it is not available in most hospitals and is still costly. It also requires an invasive technique involving the insertion of a specialized catheter.^18^ A tool for evaluating stent apposition during the procedure and accessing long-term results with wide availability is desirable. In contrast to MRI or OCT, 3D fusion imaging can be less invasively included in the routine workflow, and the fused images would be available within minutes.^8^ With the aid of commercial software, motion artifacts can be minimized through landmark-based registration between HR-CBCT and 3D-DSA images.^6^^,^^7^ Surgeons can assess stent apposition from various perspectives by manipulating the 3D fusion images on their workstations, offering an advantage over 2D images.^8^ However, the additional radiation dose to the patient is a drawback, although we believe that it is acceptable for obtaining precise information on stent apposition during LVIS deployment.

Our study has some limitations. Only a small number of cases from a single center were included and edge malapposition was rare. Of these, three quarters of the aneurysms were located in the carotid siphon, therefore we cannot generalize our results to aneurysms in other locations. The median diameter of aneurysms included in the present study was smaller than 10 mm, but larger coil masses may produce more image artifacts and make the assessment of malapposition challenging. We did not compare 3D fusion images with 2D MIP images because we did not routinely acquire CBCT images after low-concentration contrast medium administration. Some errors may have been introduced in the measurements of malapposition between the stent and vessel wall due to the manual windowing of the images. Similarly, the reconstruction parameters were not standardized, which could have potentially affected the visibility of the stent structures and vessels.^19^

## 5 Conclusion

The 3D fusion imaging technique can visualize the precise 3D structure of both the LVIS stent and the parent artery with excellent inter-rater reliability, and it allows the evaluation of stent apposition. Malapposition was detected in a part of the LVIS stents, indicating that it should not be ignored. This technique provides surgeons with a valuable tool to evaluate stent apposition in real-time during the procedure, enhancing the effectiveness of conducting stent-assisted coiling with LVIS stents.

## Funding

This study was partially supported by a research grant (ID C00221026) from 10.13039/501100004830Siemens Healthcare K.K.

## CRediT authorship contribution statement

**Naoki Kato:** Writing – original draft, Conceptualization. **Toshihiro Ishibashi:** Writing – review & editing, Supervision, Investigation, Data curation, Conceptualization. **Katharina Otani:** Writing – review & editing, Formal analysis, Data curation. **Yukiko Abe:** Methodology, Data curation. **Tohru Sano:** Writing – review & editing, Investigation, Data curation. **Gota Nagayama:** Writing – review & editing, Validation, Data curation. **Michiyasu Fuga:** Writing – review & editing, Validation, Data curation. **Shunsuke Hataoka:** Writing – review & editing, Supervision, Data curation. **Issei Kan:** Writing – review & editing, Validation, Supervision. **Yuichi Murayama:** Writing – review & editing, Supervision, Funding acquisition, Conceptualization.

## Declaration of competing interest

K.O. is an employee of Siemens Healthcare. The other authors declare no conflicts of interest.

## Abbreviations and acronyms

CBCT: cone beam computed tomography
DSA: digital subtraction angiography
HR: high resolution
ICA: internal carotid artery
IRB: institutional review board
LVIS: low-profile visualized intraluminal support
MIP: maximum intensity projection
MRI: magnetic resonance imaging
OCT: optical coherence tomography
3D: three dimensional
2D: two dimensional
VER: volume embolization ratio
